# Comment on Alfassam et al. Development of a Colorimetric Tool for SARS-CoV-2 and Other Respiratory Viruses Detection Using Sialic Acid Fabricated Gold Nanoparticles. *Pharmaceutics* 2021, *13*, 502

**DOI:** 10.3390/pharmaceutics14091871

**Published:** 2022-09-05

**Authors:** Milad Zandi, Saber Soltani

**Affiliations:** Department of Virology, School of Public Health, Tehran University of Medical Sciences, Tehran 1417613151, Iran

**Keywords:** SARS-CoV-2, MERS-CoV, hemagglutinin

## Abstract

In a published article in *Pharmaceutics*, researchers developed a sialic acid (SA) stabilized Au nanoparticle system based on SA’s binding ability that exists on the surface of lungs epithelial cells. The authors reported that many respiratory viruses including influenza, Middle-East respiratory syndrome (MERS-CoV), and the current coronavirus (SARS-CoV-2) bind to SA as one of the main binding targets of the surface protein hemagglutinin (HA).

In a published article in *Pharmaceutics*, researchers developed a sialic acid (SA) stabilized Au nanoparticle system based on SA’s binding ability that exists on the surface of lungs epithelial cells [1]. The authors reported that many respiratory viruses including influenza, Middle-East respiratory syndrome (MERS-CoV), and the current coronavirus (SARS-CoV-2) bind to SA as one of the main binding targets of the surface protein hemagglutinin (HA) [1]. Viruses in lineage A of the genus betacoronavirus, such as human coronaviruses OC43-CoV and HKU1-CoV, and bovine coronavirus attach to 9-*O*-acetylated sialoglycans using a spike protein with hemagglutinin-esterase (HE) acting as a receptor-destroying enzyme [2]. In bovine coronavirus, the HE lectin domain stimulates esterase activity toward clustered substrates. However, OC43-CoV and HKU1-CoV have lost the function of HE lectin as an adaptation in humans [2]. The authors also stated that HA is located on the surface of SARS-CoV-2 [1]. However, according to scientific evidence, SARS-CoV-2 like SARS-CoV as a betacoronavirus of lineage B, lacks hemagglutinin and hemagglutinin-esterase [3,4,5,6,7]; therefore, spike proteins of SARS-CoV-2 bind to cell surface sialic acid molecules. They reported SA can be used as a binding site for the viral surface protein HA in SARS-CoV-2 and other viral types, such as influenza and MERS-CoV viruses [1]. In addition, according to the claim of authors, influenza B, MERS-CoV, and SARS-CoV-2 viruses can modify various host specificities depending on their HA glycoprotein that presents on the viral surface [1]; however, according to scientific evidence no member of the coronaviruses in *Coronaviridae* family has HA glycoprotein. Thus, HA cannot be used for binding to SA in SARS-CoV-2 and MERS-CoV.

The family *Coronaviridae* of the *Nidovirales* order is classified into four genera: α-CoV, β-CoV, γ-CoV, and δ-CoV. β-CoV is subdivided into five different subgenus including Embecovirus (known as lineage A), Sarbecovirus (lineage B), Merbecovirus (lineage C), Nobecovirus (lineage D), and Hibecovirus [8]. SARS-CoV and SARS-CoV-2 are β-CoVs of lineage B; OC43-CoV and HKU1-CoV are considered as lineage A β-CoVs; and MERS-CoV is considered as a β-CoV of lineage C [9,10].

Among coronaviruses, β-CoVs of lineage A such as OC43-CoV and HKU1-CoV harbor the hemagglutinin-esterase (HE) gene not HA [10]. The transmission of HE gene to lineage A β-CoVs progenitor has happened via horizontal gene transfer from influenza virus C/D as a form 9-*O*-Ac-SA–specific hemagglutinin-esterase-fusion (HEF) [10]. The HE in some β-CoVs does not have membrane fusion activity unlike HEF, but they are an accessory to the spike protein S [11].

The SARS-CoV-2 and MERS-CoV genomes lack HE gene [10,11,12] as shown in Figure 1. Thus, these viruses cannot encode HE and use spike glycoprotein to bind sialic acid. The spike glycoprotein contains two subunits including S1 and S2. The receptor-binding domain (RBD), which is located in the S1 subunit, is responsible for binding to the receptor, and the S2 subunit induces the viral fusion and entry process into the host cell. Therefore, SARS-CoV-2, MERS-CoV can bind to SA using the spike glycoprotein.

## Figures and Tables

**Figure 1:**
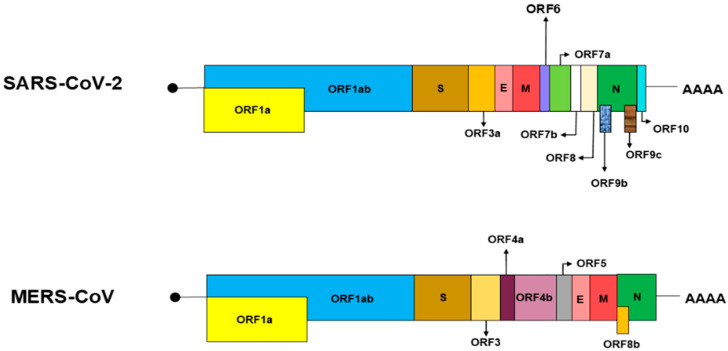
Structure of SARS-CoV-2 and MERS-CoV genomes.
